# Effect of laser-microtexturing on bone and soft tissue attachments to dental implants: A systematic review and meta-analysis

**DOI:** 10.34172/joddd.2021.048

**Published:** 2021-12-05

**Authors:** Roodabeh Koodaryan, Ali Hafezeqoran

**Affiliations:** Dental and Periodontal Research Center, Tabriz University of Medical Sciences, Tabriz, Iran

**Keywords:** Dental implant, Implant collar, Laser microtexturing, Marginal bone loss, Meta-analysis

## Abstract

**Background.** It is critical to understand laser-microtextured implant collars’ influence on peri-implant pocket depths and marginal bone levels, especially in crucial areas. The present review investigated the peri-implant marginal bone loss (MBL) and pocket depths and failure rates of dental implants with laser-microtextured collars.

**Methods.** An electronic search was run in the PubMed and Embase databases until September 15, 2019. Randomized and prospective clinical studies comparing peri-implant MBL and pocket depths and failure rates between implants with laser-microtextured and machined collar surfaces were included. Five studies (two cohort studies and three RCTs) were included in the meta-analysis after the inclusion and exclusion criteria and qualitative assessments were applied. The risk ratio of osseointegrated implant failure and mean differences in peri-implant MBL and pocket depths were calculated using the Comprehensive Meta-Analysis (CMA) software.

**Results.** Implants with laser-microtextured collars exhibited significantly better marginal bone level scores (*P* < 0.001; MD: 0.54; 95% CI: 0.489‒0.592) and a significant reduction in peri-implant probing depths than implants with machined collars (*P* < 0.001; MD: 1.01; 95% CI: 0.90‒1.13). The assessed studies showed that 17 out of 516 implants failed (3.29%), comprising nine implants with machined (3.62%) and eight implants with laser-microtextured collars (2.98%). However, no significant differences were detected in the implant neck surface characterization (*P* = 0.695; RR: 1.205; 95% CI: 0.472‒3.076).

**Conclusion.** This study suggests that laser-microtexturing of implant collar significantly affected the peri-implant MBL and probing depths. Although no significant differences were noted in implant failure rates between implants with laser-microtextured and machined collar surfaces, the peri-implant MBL and probing depths with laser-microtextured collars were significantly lower than the machined collars.

## Introduction

Peri-implant soft tissues and restorative measures are widely used to assess implant dentistry outcomes.^1^ These outcome measures are associated with soft tissue stability and concurrently affect the crestal bone level changes. Marginal bone loss (MBL) is a critical factor for predictable and long-term esthetic and health results. The peri-implant bone loss might cause pocket formation; therefore, peri-implant tissue health of osseointegrated implants and survival can be adversely affected.^2^ An MBL of 1.5 mm in the first year of function and a bone loss of 0.2 mm yearly in subsequent years have long been assumed to be perquisites for implant treatment success.^3,4^

The mechanisms responsible for peri-implant crestal bone loss are not fully understood. A variety of etiologic factors, including implant design, poor bone quality, bone characteristics, traumatic implant surgery, occlusal overload, implant‒abutment connection, diminished blood supply, periodontal status, and smoking habits might contribute to crestal bone loss.^5–7^ Some recent developments in implant neck surface treatment have led to improvements in hard and soft tissue integration and peri-implant marginal bone preservation.^8^ One of these strategies is microtexturing the dental implant collar with 8‒12-µm microgrooves using laser beams.^9^ Tissue culture studies have revealed osteoblast and fibroblast cellular attachment on laser-microtextured collars.^10,11^ These observations have been confirmed in animal and human studies.^9,12^ A direct and physical connective tissue attachment is formed on laser-ablated microgrooves with fibers oriented in a predominantly perpendicular pattern to the implant surface,^9^ which significantly differs from the fibrous capsule formed around the conventional osseointegrated implants with fibers exhibiting parallel and circumferential orientation relative to the collar surface.^13^ Thus, it has been speculated that the fibro-collagenous physical attachment around laser-microtextured collar surfaces might stabilize the bone and reduce crestal bone resorption.

The effects of laser-microtextured collar surface on marginal bone level changes and probing depths are currently unclear. Therefore, this systematic review was undertaken to compare the peri-implant MBL, probing pocket depth (PPD) and failure rates (FR) of laser-microtextured implants.

*The null hypothesis:* There is no difference in FR, MBL, and PPD around the laser-microtextured implants compared to machined ones.

## Methods

This study adhered to the PRISMA statement criteria.^14^ Based on the PICO criteria (patient, intervention, comparison, and outcome), a structured question was designed for the study as follows: For patients needing implant treatment (P), will the laser-microtextured implant collar (I) compared with machined collar (C) change the MBL, PPD around implants, and SR (O)?

### Search strategy

An electronic search was performed in the PubMed and Embase databases for potentially relevant publications until September 15, 2016. Medical Subject Headings (MeSH) terms were used as follows: Dental implant, oral implant, tooth implant, and teeth implant, combined with the following words: neck, design, laser microtexture, and Laser-Lok connected with OR and AND. An electronic search was complemented with a manual search of journals, including *British Journal of Oral and Maxillofacial Surgery, Clinical Implant Dentistry and Related Research, Clinical Oral Implants Research, European Journal of Oral Implantology, Implant Dentistry, International Journal of Oral and Maxillofacial Implants, International Journal of Oral and Maxillofacial Surgery, International Journal of Periodontics and Restorative Dentistry, International Journal of Prosthodontics, Journal of Clinical Periodontology, Journal of Dental Research, Journal of Dentistry, Journal of Oral Implantology, Journal of Craniofacial Surgery, Journal of Cranio-Maxillofacial Surgery, Journal of Maxillofacial and Oral Surgery, Journal of Oral and Maxillofacial Surgery,*and* Journal of Periodontology.*

### Eligibility criteria

Randomized clinical trials (RCTs) and controlled clinical trials (CCTs) comparing the MBL, PPD, and FR between implants with laser-microtextured and machined collar surfaces were included in the systematic review. The exclusion criteria were case reports, retrospective studies, computational studies, animal studies, in vitro studies, review papers, studies evaluating only one collar surface type, and short-term follow-up periods (<1 year).

### Study selection

The titles were initially screened by two authors independently. The studies’ abstracts were screened, and those meeting the inclusion criteria underwent further evaluations. Besides, the reference lists of the studies selected were scanned for more publications. Any disagreements between the authors were resolved through discussion to reach an agreement.

### Quality assessment

All the studies were quality-assessed by using the Newcastle-Ottawa scale (NOS).^15^ This scale calculates the potential risk of individual studies bias based on three major components: selection, comparability, and outcome for cohort studies. The NOS assigns a maximum of four, two, and three stars for selection, comparability, and outcome, respectively. Studies with a score of ≥6 on NOS (maximum score = 9) were considered of high methodological quality. NOS scores ≤4 were considered to have a high bias risk.

### Data extraction and meta-analysis

The following information was extracted from the included studies in the final analysis: the year of publication, study design, implant system, failed/placed implants, patient’s age, follow-up, and peri-implant MBL and probing depths. The authors were contacted for missing data. The implant failure rate was the dichotomous and MBL, and peri-implant probing depths were the continuous outcome measures evaluated.

The risk ratio of osseointegrated implant failure and mean differences of peri-implant MBL and pocket depths were calculated with a 95% confidence interval (CI), and statistical significance was set at 5% (α = 0.05). The I^2^ index was used to quantify the proportion of total variation in estimates due to heterogeneity rather than chance. The meta-analysis was carried out with inverse variance methods. If statistically significant heterogeneity was observed among the study groups, the analysis was performed using a random-effects model. A fixed-effects model assessed the significance of treatment effects, revealing no significant heterogeneity. The data were analyzed with CMA 2.0 (Comprehensive Meta-Analysis) software (Biostat Inc., Englewood, New Jersey, USA).

## Results

**Figure 1:**
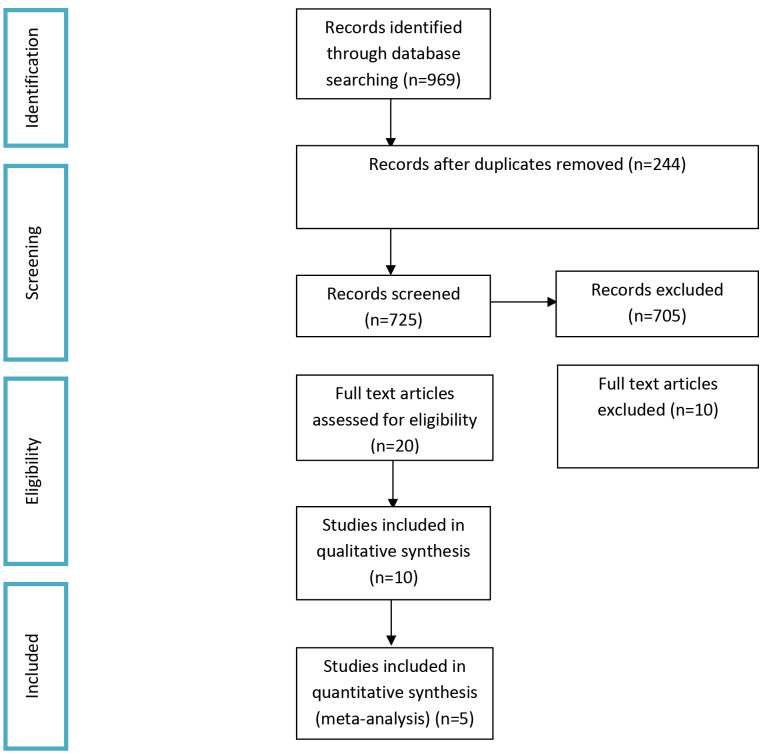
Diagram of the search strategy.

The databases’ search strategy resulted in 969 papers, including 880 from PubMed and 89 from Embase. The duplicates were identified; then, the authors screened the abstracts independently. Initial screening retrieved 10 publications (five cohort studies, two retrospective studies, and three RCTs) (Figure 1). However, after applying the inclusion and exclusion criteria and the selected studies’ qualitative assessment, five studies^16-20^ (two cohort studies and three RCTs) remained for the meta-analysis (Table 1). The kappa inter-investigator agreement was 0.98 for studies from the PubMed and 0.91 for studies from the Embase, indicating a high level of agreement.

**Table 1:** Detailed data of the included studies

**Authors**	**Published**	**Study design**	**Patient’s age range (average) (year)**	**Follow-up visits** **(month)**	**Implant system**	**Implant design surface**	**Collar surface**	**Failed/placed implant (n)**	**MBL (mm)**	**PPD (mm)**
Botos^19^	2011	CCT	40-74 (57)	6, 12	Biolok Internastional Noble Biocare	-	LM	1/30	0.42 ± 0.34	0.43 ± 0.51
						-	M	1/30	1.13 ± 0.61	1.64 ± 0.93
Guarnieri^20^	2014	CCT	43-75 (49.3	6, 12, 24	BioHorizon	Tapered, Internal, RBT	LM	4/160	0.58 ± 0.17	-
							M	5/140	1.09 ± 0.37	-
Farronato^18^	2014	RCT	45-65 (49.3)	6,12,24	BioHorizon	Tapered, Internal, RBT	LM	1/39	0.49 ± 0.34	-
							M	1/39	1.07 ± 0.30	-
Guarnieri^17^	2015	RCT	45-65 (49.3)	36	BioHorizon	Tapered, Internal	LM	2/39	0.65 ± 0.22	0.84 ± 0.37
							M	2/39	1.24 ± 0.28	1.81 ± 0.18
Guarnieri^16^	2016	RCT	NA (57.1)	12	BioHorizon	Tapered, Internal, RBT	LM	0/17	0.19 ± 0.06	1.31 ± 0.51
							M	0/17	0.35 ± 0.17	2.66 ± 0.83

RBT: Resorbable Blast Texturing, NA: not available, MBL: marginal bone loss, PPD: peri-implant bone loss

Five studies were included in this quantitative meta-analysis, published from 2011 to 2016. Three RCTs and four CCTs were included in the meta-analysis. All the studies included only adult patients aged 40‒74 years. All the studies assessed two types of implant collars (laser microtextured or machined collar), surface dimension, connection type, and with comparable macro-design (tapered implants). A total of 550 implants were evaluated, of which 285 implants were laser-microtextured, and 265 had machined collar. The follow-up period range was 1‒3 years. One study investigated the outcome of MBL when different implant placement protocols (immediate or delayed) and loading (immediate non-occlusal or delayed loading) were used.^21^ In another study, the implants were inserted in periodontally compromised patients with a nonsurgical treatment history.^22^ In one study, the patients received mandibular implant-supported overdentures,^19^ whereas the rest of the implants were prosthetically restored with single crowns.

Radiographic assessment of MBL was performed using standardized digital intraoral radiographs. In all the studies, periapical radiographs with custom-made radiograph holders furnished an estimate of changes at the follow-up intervals.

Table 2 presents the risk of bias in each study. Four studies^16-19^ were considered high quality, and one^20^ was deemed moderate quality.

**Table 2:** Quality assessment of the studies by the Newcastle-Ottawa scale

**Study**	**Published**	**Selection**			**Comparability of cohorts**			**Outcome**			**Total (9/9)**
		**Representativeness of the exposed cohort**	**Selection of external control**	**Ascertainment of exposure**	**Outcome of interest not present at start**	**Main factor**	**Additional factor**	**Assessment of outcome**	**Follow-up long enough** ^a^	**Adequacy of follow-up**	
Botos^19^	2011	*	*	*	*	*	0	*	0	0	6/9
Guarnieri^20^	2014	0	*	*	*	*	0	*	0	0	5/9
Farronato^18^	2014	*	*	*	*	*	*	*	0	0	7/9
Guarnieri^17^	2015	*	*	*	*	*	*	*	0	0	7/9
Guarnieri^16^	2016	*	*	*	*	*	*	*	0	0	7/9

^a^Five years was chosen to be enough for the outcome ‘implant failure’ to occur.

### Marginal bone loss

**Figure 2:**
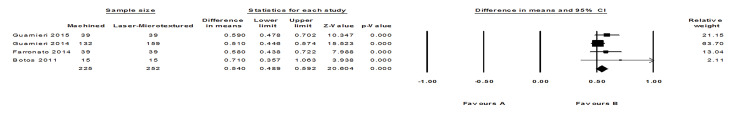
Forest plot for the event ‘marginal bone loss’ in the comparison between machined and laser-microtextured neck implants.

**Figure 3:**
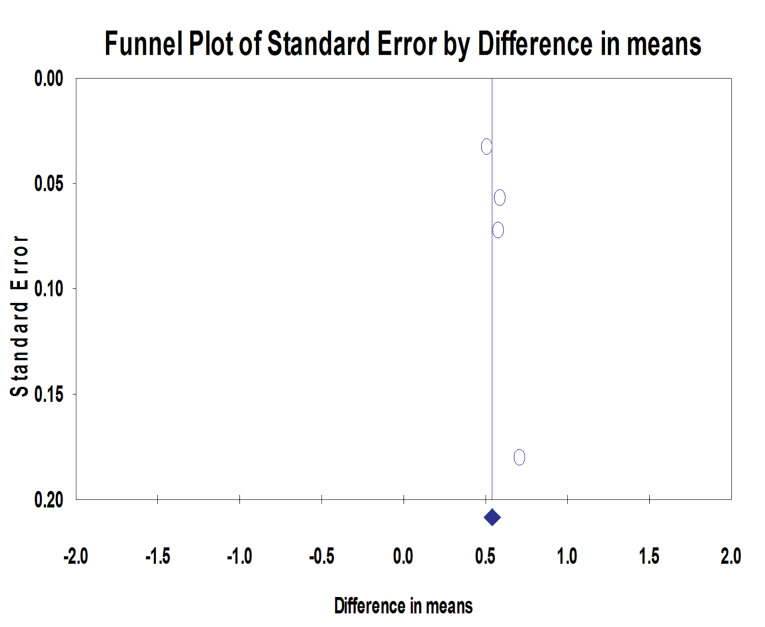
Funnel plots for the studies reporting the outcome event ‘marginal bone loss’.

Four studies assessed the mean peri-implant marginal bone changes (mm) at different follow-up intervals. The MBL range was 1.07‒1.24 mm in the machined neck group and 0.42‒0.65 mm in the laser-microtextured implant neck group. Implants with laser-microtextured collars exhibited significantly less MBL than machined-neck implants (*P* < 0.001; MD: 0.54; 95% CI: 0.489‒0.592) (Figure 2). Tests for homogeneity were not significant (*P* = 0.424), suggesting homogeneity among these studies (I^2^ = 0%). The symmetrical funnel plot revealed no evidence of publication bias (Figure 3).

### Peri-implant probing depth

**Figure 4:**
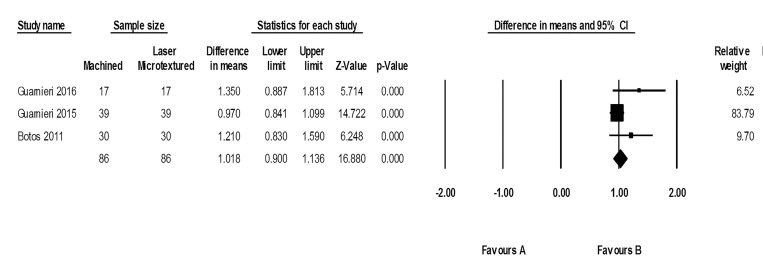
Forest plot for the event ‘peri-implant probing depth’ in the comparison between machined and laser-microtextured neck implants.

**Figure 5:**
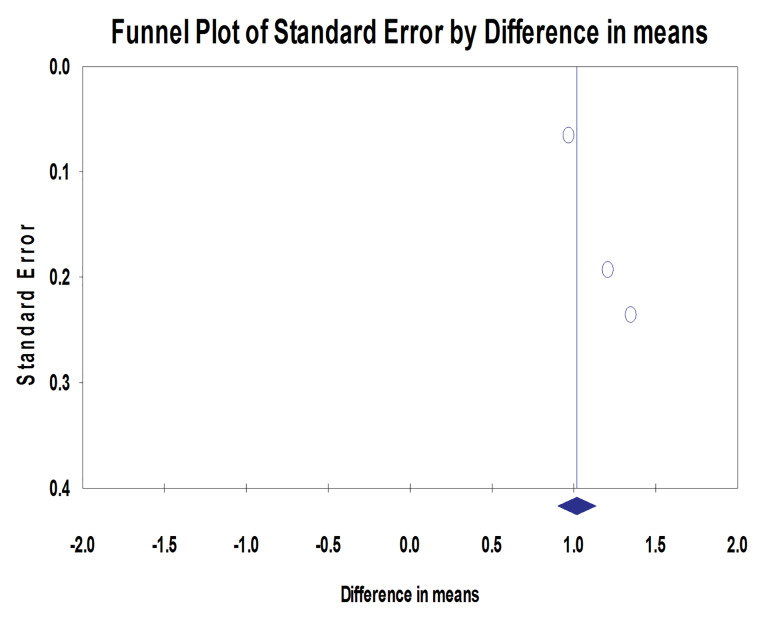
Funnel plots for the studies reporting the outcome event ‘peri-implant probing depth’.

The results of the three studies were combined for data synthesis. The peri-implant pocket depth range was 1.64–2.66 mm in machined neck groups, with 0.84–1.31 mm in laser-microtextured neck implants. Implants with laser-microtextured collars exhibited significantly less PPD than machined-neck implants (*P* < 0.001; MD: 1.01; 95% CI: 0.90‒1.13) (Figure 4). Tests for homogeneity were not significant (*P* = 0.175), suggesting acceptable heterogeneity among these studies (I^2^ = 42.66%). The funnel plot did not reveal apparent asymmetry (Figure 5).

### Implant failure rates

**Figure 6:**
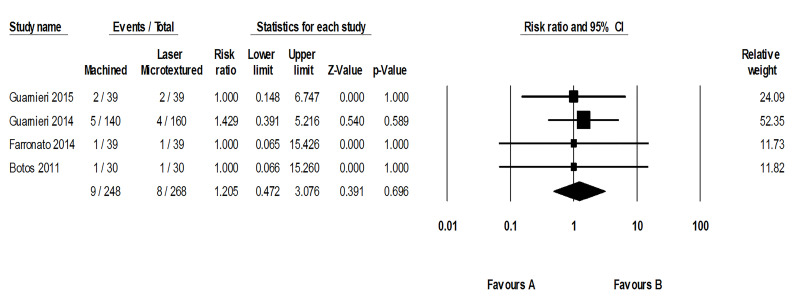
Forest plot for the event ‘implant failure rate’ in the comparison between machined and laser-microtextured neck implants

**Figure 7:**
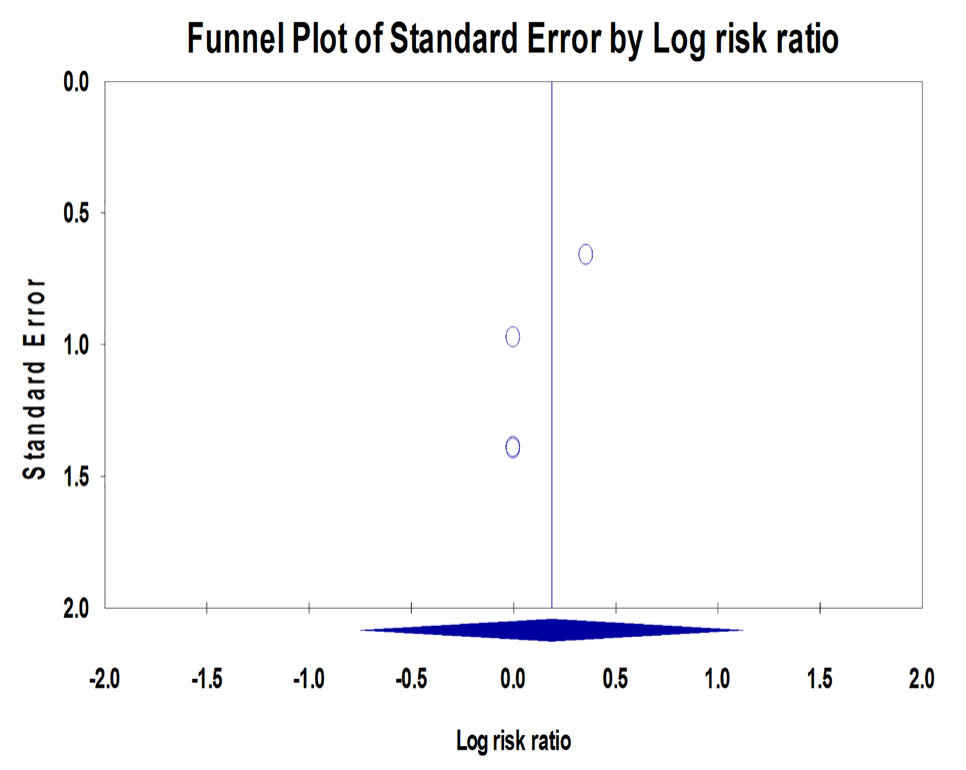
Funnel plots for the studies reporting the outcome event ‘implant failure rate’.

In the assessed studies, 17 out of 516 implants failed (3.29%), comprising nine machined-neck implants (3.62%) and eight laser-microtextured implants (2.98%) (Figure 6). Quantitative analysis revealed no significant difference due to the implant neck surface characterization (*P* = 0.695; RR: 1.205; 95% CI: 0.472‒3.076). Tests for homogeneity (*P* = 0.987) suggest homogeneity among the studies (I^2^ = 0%). The symmetrical funnel plot revealed no evidence of publication bias (Figure 7).

## Discussion

The current meta-analysis was designed to compare the MBL, PPD, and FR in implants with laser-microtextured collar surfaces. Data synthesis showed that MBL and PPD around implants with laser-microtextured collar surfaces were significantly less than the machined collars, suggesting that laser-microtextured implants might provide better outcomes than machined ones.

Stable bone level around dental implants is a crucial criterion for long-term implant survival and affects esthetic outcomes. Marginal bone level change of 1–1.5 mm in the first year of function, followed by an annual bone loss of 0.2 mm after that, is considered a successful treatment.^3^ Several studies have assessed the impact of implant macro- and microstructure on the distribution of mechanical stresses between the implant’s coronal portion and the surrounding bone.^8,23-26^ However, the effect of the collar surface on MBL has recently attracted attention. Studies suggest that the addition of bone retentive features, including microthreads and microgrooves at the coronal portion of an implant, might give rise to a larger bone–implant contact and might thus be a possible means of preserving marginal bone level.^23,25,27-29^ Using a 3D finite element analysis, Hansson hypothesized that the biomechanical interlocking capacity of these elements with bone increases the interfacial shear strength and the resistance of crestal bone to resorption,^30^ which was substantiated by some recent clinical studies that demonstrated a decrease in marginal bone changes around rough-surfaced microthreaded collars compared to machined and rough-surfaced implants.^31-34^

Laser microtexturing of implant collar surface has been investigated in several in vitro and in vivo studies. The controlled laser ablation technology creates surface microchannels that might allow a direct connective tissue attachment to implant and abutment surfaces.^9,12^ Several clinical and histological studies have confirmed that the laser-ablated retentive features favorably affect bone stability during the early phase of implant treatment and thus reduce MBL.^35^ The present meta-analysis results revealed a significant difference between the laser-microtextured and machined implants in MBL (MD: 0.54; CI: 0.489–0.592, *P* < 0.001). The higher marginal bone attachment to this microgeometry would be reasonable considering the significant effect the substrate can exert on cell growth and development.^36^ In vitro tissue culture studies have demonstrated that fibroblast and osteoblast precursors exhibit different attachment, growth, spreading, and orientation in the functional laser-microgroove layout.^11,37^ Accordingly, microgrooves on the collar surface might control hard and soft tissues’ responses to implant materials and provide a predetermined site to establish a physical connective tissue attachment.

Previous studies have shown improvements in periodontal probing depths around laser-microtextured implants compared to machined collars, indicating that a soft tissue seal is established on the bone at implant sites. Concerning clinical parameters, the present meta-analysis results confirm those of previous studies. The laser-microtextured collar resulted in a significant reduction in peri-implant probing depth than machined implants (*P* < 0.001; MD: 1.01; 95% CI: 0.90–1.13).

A critical issue is the presence of several limitations. First, the present meta-analysis included a limited number of published studies with short follow-up periods. Only one study^17^ followed MBL three years after functional loading. Farronato et al^18^ and Guarnieri et al^21^ observed MBL at 6, 12, and 24 months of follow-up, and Botos et al^19^ at 6 and 12 months of follow-up. A longer follow-up might have led to a more significant increase in the failure rate. Implant-supported prostheses might be affected by several external and internal forces after functional loading; thus, the real failure rate might be underestimated. Second, differences in the prosthetic design must be taken into account. Four studies^16-18,20,22^ rehabilitated the patients with fixed prostheses, and only one^19^ was an implant-supported overdenture. Third, MBL depends on several factors, and microgrooves are only one of them. Other factors that influence the marginal bone level are grafting, implant insertion in fresh sockets, healing period lengths, occlusion of the opposite arch, implant angulation, loading protocol, and bone type. Considering these limitations, more RCTs with more extended follow-up periods are required to determine the real effect of laser-microtextured collar surfaces on marginal bone maintenance.

## Conclusion

The following conclusions were drawn under the limitations of the current study:

1. MBL and probing depths around implants with a laser-microtextured collar were significantly less than the machined collars. However, due to the limited data available in the literature, the evidence was insufficient, necessitating further RCTs with more extended follow-up periods.

2. No significant differences were detected in implant failure rates between implants with laser-microtextured and machined collar surface.

## Authors’ Contributions

Both authors contributed in design of the study, data search, manuscript preparation and revision.

## Acknowledgments

None.

## Funding

Not applicable.

## Competing Interests

The authors declare no competing interests with regards to the authorship and/or publication of this article.

## Ethics Approval

Not applicable.
